# Tuning Thermal Transport in Radio-Frequency-Sputtered Beta-Gallium Oxide Through Thickness and Plasma-Induced Interface Modification

**DOI:** 10.3390/nano16211439

**Published:** 2026-11-06

**Authors:** Azat Abdullaev, Rustem Tlegenov, Magomed A. Umakhanov, Jacques O’Connell, Abubakar M. Ismailov, Kairolla Sekerbayev, Zhandos N. Utegulov

**Affiliations:** 1 Center for Energy and Advanced Materials Science, National Laboratory Astana, Astana 010000, Kazakhstan; rustem.tlegenov@nu.edu.kz (R.T.); kairolla.sekerbayev@nu.edu.kz (K.S.); 2 Department of Physics, School of Sciences and Humanities, Nazarbayev University, Astana 010000, Kazakhstan; 3 Institute of New Materials and Energy Technologies, 53 Kabanbay Batyr Ave., Astana 010000, Kazakhstan; 4 Department of Physical Electronics, Faculty of Physics and Technology, Dagestan State University, Makhachkala 367000, Russia; umagomed.1999.3@gmail.com (M.A.U.); egdada@mail.ru (A.M.I.); 5 Centre for HRTEM, Nelson Mandela University, Port Elizabeth 6001, South Africa; jacques.oconnell@gmail.com

**Keywords:** beta-gallium oxide, thermal conductivity, thermal boundary conductance, radio-frequency magnetron sputtering, plasma treatment, time-domain thermoreflectance, frequency-domain thermoreflectance

## Abstract

Controlling heat flow in beta-gallium oxide (β-Ga_2_O_3_) thin films requires understanding both phonon transport within the semiconductor and heat transfer across metal/semiconductor interfaces. Here, we investigate these pathways in high-temperature radio-frequency magnetron-sputtered β-Ga_2_O_3_ films grown on c-plane sapphire with thicknesses from 0.42 to 2.1 μm. The thermal conductivity increases from 5.4–6.9 W m^−1^ K^−1^ in the 420 nm film to approximately 10–12 W m^−1^ K^−1^ in the thicker films. Structural characterization reveals thickness-dependent microstructural evolution, including grain coarsening and intergranular void-like defects. An analytical Debye–Klemens model incorporating phonon scattering from film boundary and defects reproduces the overall thickness dependence. The Ar plasma-treated samples exhibit Al/β-Ga_2_O_3_ thermal boundary conductance values of approximately 100 MW m^−2^ K^−1^, about 70% higher than the untreated sample (~60 MW m^−2^ K^−1^), while the thermal conductivity of the underlying films remains within a comparable range. Picosecond acoustic measurements, transmission electron microscopy and electron energy loss spectroscopy reveal a corresponding modification of the interfacial acoustic response and formation of an ultrathin amorphous/disordered near-surface region. Although variations between independent deposition runs cannot be excluded, these findings suggest that thickness-dependent microstructure and plasma-associated interface modifications offer promising approaches to tailoring thermal transport in β-Ga_2_O_3_-based devices.

## 1. Introduction

The development of high-power electronics has driven increasing interest in ultrawide-bandgap (UWBG) semiconductors capable of sustaining high electric fields and power densities. Among these materials, beta-gallium oxide (β-Ga_2_O_3_) has emerged as a promising candidate for next-generation power electronics owing to its ultrawide bandgap of approximately 4.8 eV, high critical electric field exceeding 8 MV/cm, and the availability of large-area single-crystal substrates produced by melt-growth techniques [1,2,3]. These characteristics make β-Ga_2_O_3_ attractive for high-voltage switches, power diodes, and other electronic devices operating under high electric-field and high-power-density conditions.

Despite these favorable electronic properties, thermal transport is a major limitation for β-Ga_2_O_3_-based electronics. The thermal conductivity of β-Ga_2_O_3_ is relatively low and strongly anisotropic, with reported values of approximately 10–30 W m^−1^ K^−1^ depending on crystallographic direction, substantially below those of established wide-bandgap semiconductors such as GaN and SiC [4,5,6]. The combination of high power density and low thermal conductivity can result in substantial self-heating near the active region, increasing device operating temperature and potentially degrading performance and reliability [7,8]. Consequently, understanding and controlling heat transport in β-Ga_2_O_3_ is essential for realizing the full potential of its electronic properties.

For thin films, however, the relevant thermal conductivity can differ substantially from the intrinsic bulk value. Previous studies have demonstrated pronounced thickness- and microstructure-dependent thermal transport in β-Ga_2_O_3_ films prepared by different growth and transfer approaches [9,10,11,12,13,14]. Finite film thickness limits the mean free path of heat-carrying phonons through boundary scattering, while grain boundaries, point defects, strain, implantation-induced damage and other structural imperfections can provide additional phonon scattering channels. Recent studies have also demonstrated that engineering the arrangement of extended defects offers an additional means of tailoring phonon transport [15]. Accordingly, mechanically exfoliated single-crystal films generally exhibit increasing thermal conductivity with increasing thickness, approaching the corresponding bulk value, whereas deposited or defect-rich films often exhibit substantially lower thermal conductivities [9,10,11,12,13,14]. Establishing the relationship between film thickness, microstructure, and thermal conductivity is therefore important for realistic thermal design of β-Ga_2_O_3_ thin-film devices.

Radio-frequency (RF) magnetron sputtering is an attractive approach for β-Ga_2_O_3_ thin-film growth because of its relative simplicity, scalability, thickness control, and compatibility with large-area substrates. Previous studies have shown that the crystalline quality, preferred orientation, surface morphology, stoichiometry, and optoelectronic properties of RF-sputtered Ga_2_O_3_ are strongly influenced by deposition parameters such as substrate temperature, sputtering pressure, RF power, and substrate orientation [16,17,18,19]. In particular, optimization of the sputtering pressure and gas composition can substantially modify film crystallinity and surface morphology, while high-temperature RF sputtering has recently enabled highly crystalline β-Ga_2_O_3_ growth on sapphire substrates [17,19]. Nevertheless, compared with the extensive structural, optical, and electrical characterization of RF-sputtered β-Ga_2_O_3_, its thermal transport properties and their relationship with thickness-dependent microstructural evolution remain largely unexplored.

Thermal management, however, extends beyond heat transport within the semiconductor itself. In practical transistors and power diodes, heat generated near the active region must be dissipated across multiple interfaces, including those between β-Ga_2_O_3_ and metal contacts or thermal-management layers. In particular, metal contacts positioned near high-temperature regions of a device can provide an additional heat-removal pathway when their thermal boundary conductance (TBC) is sufficiently high [7,20]. Previous studies have demonstrated that thermal transport across metal/β-Ga_2_O_3_ interfaces is highly sensitive to interfacial structure, bonding, and chemical composition [20,21]. More recently, it was demonstrated that surface pretreatment and deposition conditions can substantially modify thermal conductance across Al/nonmetal interfaces by altering interfacial bonding strength, while leaving the thermal conductivity of the underlying material largely unaffected [22]. However, despite previous investigations of metal/β-Ga_2_O_3_ interfaces, the potential of plasma-induced surface modification to enhance their thermal boundary conductance remains insufficiently explored. In particular, understanding how plasma-induced near-surface structural changes influence interfacial heat transfer is important for developing effective thermal management strategies in β-Ga_2_O_3_-based electronic devices.

To address these gaps, we investigate the thermal transport properties of high-temperature RF magnetron-sputtered β-Ga_2_O_3_ thin films over a range of thicknesses and correlate the measured thermal transport with their structural and morphological characteristics. We further demonstrate the relationship between Ar plasma surface treatment and the thermal boundary conductance of an Al/β-Ga_2_O_3_ interface. By combining thermal measurements with electron microscopy, surface morphology characterization, picosecond acoustic measurements and interface chemistry analysis, we establish a relationship between plasma-induced surface/interface modification and thermal transport across the Al/β-Ga_2_O_3_ interface. These results highlight the importance of both film microstructure and near-surface interface engineering for controlling thermal transport in sputtered β-Ga_2_O_3_ thin films.

## 2. Materials and Methods

### 2.1. Sample Preparation

β-Ga_2_O_3_ thin films were deposited on (0001)-oriented c-plane sapphire substrates by high-temperature radio-frequency magnetron sputtering and hereafter referred to as RF-sputtered films, following the deposition protocol developed in our previous work [19]. Only the key growth parameters relevant to the present study are summarized here. A ceramic Ga_2_O_3_ target fabricated from 99.999% pure starting material was sputtered at an RF frequency of 13.56 MHz in a reactive Ar/O_2_ atmosphere with an Ar:O_2_ ratio of 1:1. The total working pressure was 1.07 Pa, the applied RF power was 100 W, and the target-to-substrate distance was 5 cm.

The use of RF magnetron sputtering was dictated by the electrically insulating nature of the Ga_2_O_3_ ceramic target, which makes conventional DC sputtering unsuitable due to surface charge accumulation. The frequency of 13.56 MHz corresponds to a standard ISM operating frequency commonly used in RF sputtering systems. The selected deposition parameters correspond to the experimentally validated growth conditions reported in Ref. [19], which enabled reproducible epitaxial growth of β-Ga_2_O_3_ films on c-plane sapphire substrates.

To enable efficient high-temperature heating of the infrared-transparent sapphire substrates, an approximately 1 μm-thick Ta layer was deposited on the backside of each substrate prior to β-Ga_2_O_3_ growth. The Ta coating enhanced absorption of thermal radiation from the heater and enabled stable heating of the sapphire substrates at the temperatures required for high-temperature growth. The β-Ga_2_O_3_ films investigated in this study were deposited at a substrate temperature of 1200 °C without post-growth annealing and had thicknesses ranging from approximately 0.42 to 2.1 μm.

The reproducibility of the deposition procedure was established in our previous study through multiple independent growth runs performed under identical nominal conditions. These experiments demonstrated reproducible structural and morphological characteristics across the sample series, while the substrate temperature was maintained within ±5 °C during deposition [19].

To investigate the effect of plasma-induced surface modification on thermal transport, three β-Ga_2_O_3_ samples, denoted S1, S2, and S3, were prepared in separate deposition runs under the same nominal growth conditions. Thus, the samples represented independently grown specimens produced using the same reproducible deposition protocol. Sample S1 was retained as an untreated reference, whereas samples S2 and S3 were subjected to RF Ar-plasma sputter treatment in the same magnetron sputtering system.

For the plasma-sputtering treatment, each sample was placed directly on the surface of the same 2-inch Ga_2_O_3_ ceramic target used for film deposition, with the β-Ga_2_O_3_ film surface exposed to the discharge. The annular erosion track of the magnetron target had an approximate radial width of 15 mm, whereas the lateral dimension of the sample was approximately 10 mm. Thus, the entire sample was positioned within the target erosion-track region, where intense ion bombardment and sputtering occur during RF discharge operation. The remaining uncovered portions of the target continued to be sputtered during treatment. The process was carried out in pure Ar at a working pressure of 1.07 Pa, without O_2_ addition. Samples S2 and S3 were treated at RF powers of 50 W for 5 min and 100 W for 2 min, respectively.

### 2.2. Structural Characterization and Surface Morphology

The structural properties of the β-Ga_2_O_3_ thin films were characterized using X-ray diffraction (XRD), Raman spectroscopy, atomic force microscopy (AFM), high-resolution transmission electron microscopy (HRTEM) and scanning electron microscopy (SEM). XRD measurements were performed in powder diffraction geometry using a Rigaku SmartLab (Rigaku Corporation, Tokyo, Japan) diffractometer with Cu Kα radiation over a 2θ range of 15°–80° to evaluate the crystalline structure and phase composition of the films. Raman spectroscopy was carried out using a Horiba LabRAM (Horiba, Kyoto, Japan) micro-Raman system with excitation provided by a 532 nm single-mode laser (Torus 532, Laser Quantum, Stockport, United Kingdom). The system was equipped with a 50× objective lens, resulting in a laser spot diameter of approximately 1 µm. The spectra were collected using an 1800 grooves/mm grating with an acquisition time of 120 s. Surface morphology and roughness were investigated by AFM using a SmartSPM 1000 (Horiba, Kyoto, Japan) scanning probe microscope. Cross−sectional TEM lamellae were produced by FIB lift-out using a Thermo FISHER Helios 650 dual beam (Thermo Fisher, Waltham, MA, USA). Imaging was done at 200 kV in a JEOL ARM 200F (JEOL Ltd., Tokyo, Japan). Electron energy-loss spectroscopy (EELS) was employed for chemical analysis using a Gatan GIF Quantum spectrometer (GATAN, Pleasanton, CA, USA), and elemental quantification was performed using Gatan DigitalMicrograph 3.62.4983.0 software. The evolution of film morphology and grain structure was further analyzed by SEM using a Crossbeam 540 microscope (Carl Zeiss Microscopy GmbH, Jena, Germany).

### 2.3. Thermal Transport Measurements

Thermal transport measurements were performed using time-domain thermoreflectance (TDTR) and frequency-domain thermoreflectance (FDTR) methods. Both setups are described in detail elsewhere [23,24,25,26]. Prior to TDTR measurements, an Al transducer layer with a thickness of approximately 80–90 nm was deposited onto the β-Ga_2_O_3_ films by magnetron sputtering. The thickness of the Al layer was determined by picosecond acoustic measurements and further confirmed by profilometry.

The TDTR system employed a Ti:sapphire mode-locked femtosecond laser (Tsunami, Spectra-Physics, Milpitas, CA, USA) operating at a wavelength of 782 nm, with a repetition rate of 80 MHz and a pulse duration of approximately 80 fs. The pump beam was modulated at a fixed frequency of approximately 10 MHz to periodically heat the Al transducer, while the time-delayed probe beam monitored the resulting thermoreflectance response. Both beams were focused and spatially overlapped on the sample surface using a 10× objective lens. The resulting beam diameter was ~13 µm, which is substantially larger than the thermal penetration depth at 10 MHz modulation frequency, providing predominantly cross-plane sensitivity. The measured ratio of the in-phase and out-of-phase lock-in signals, −V_in_/V_out_, as a function of pump–probe delay time was fitted using a multilayer heat-diffusion model [27] to extract the thermal conductivity of the β-Ga_2_O_3_ layer and the TBC of the Al/β-Ga_2_O_3_ interface.

Complementary FDTR measurements were performed using continuous-wave pump and probe lasers. Following the configuration described in our previous work [25,26], 660 nm and 785 nm lasers were used as the pump and probe beams, respectively. The pump beam was periodically modulated at the frequencies ranging over 100 kHz to 3 MHz, while the reflected probe signal was detected using a photodetector connected to a lock-in amplifier. In contrast to TDTR, where the pump–probe time delay is varied, FDTR measurements were performed by sweeping the pump modulation frequency and recording the corresponding phase lag of the thermoreflectance response. The pump and probe beams were spatially overlapped and focused onto the Al transducer using a 60× objective lens, resulting in an effective pump–probe spot size of approximately 2–3 μm, consistent with the configuration used in our previous FDTR measurements. The measured phase response as a function of modulation frequency was fitted using a multilayer heat-diffusion model to extract the effective thermal conductivity of the β-Ga_2_O_3_ films [28].

To evaluate measurement repeatability, thermoreflectance measurements were performed at 4–5 spatially separated locations on each sample. The reported thermal conductivity and TBC values represent the averages of the measurements obtained at these locations. The overall uncertainty was determined by combining the standard deviation of the spatial measurements with the uncertainty arising from the fixed parameters of the multilayer heat-diffusion model in quadrature, assuming these contributions to be independent. The latter were evaluated by propagating uncertainties in the fixed inputs of the multilayer heat-diffusion model, including volumetric heat capacities (±10%), layer thicknesses (±5%), fixed thermal conductivities (±10%), and laser beam radius (±10%). The sensitivity analysis identified the Al transducer thickness and laser beam radius as important sources of uncertainty in TDTR and FDTR, respectively, together with the fixed β-Ga_2_O_3_ heat capacity. The resulting uncertainty in the extracted β-Ga_2_O_3_ thermal conductivity was approximately 14–25%, depending on film thickness and measurement technique. Further details of the sensitivity analysis and the spatial reproducibility of the thermal measurements are provided in the Supplementary Materials.

### 2.4. Analytical Thermal Conductivity Model

To gain more physical insights of the thickness-dependent thermal conductivity of β-Ga_2_O_3_, the thermal conductivity was modeled by Debye approximation based a simple analytical phonon scattering model [29]:

(1) $$k=\frac{1}{3}\sum_{j=1}^{3}\int_{0}^{\theta_{D,j}/T}v^{2}\tau (x)C(x)\mathrm{dx},$$

where *θ_D_*_,*j*_ is the Debye temperature of different phonon branches, *τ* is the total phonon relaxation time, T is the temperature, v is the average phonon velocity (4423 m/s [4]), and *C(x)* is the lattice specific heat defined as:

(2) $$C(x)=\frac{3k_{B}}{2\pi^{2}v^{3}}(\frac{k_{B}}{\hbar }{)}^{3}T^{3}\frac{x^{4}e^{x}}{({e^{x}-1)}^{2}}$$

where *k_B_* is Boltzmann’s constant and *ℏ* is reduced Planck’s constant, *x* = *ℏω*/*k_B_T*. The sum was taken over the three main phonon branches: one longitudinal acoustic (LA) and two transverse acoustics (TA). The total relaxation time, *τ*(x), is given by Matthiessen’s rule:

(3) $$\tau^{-1}\left(x\right)=\tau_{U}^{-1}+\tau_{B}^{-1}{+\tau }_{\mathrm{PD}}^{-1}.$$

$\tau_{U}^{-1},\;\tau_{B}^{-1}$ and $\tau_{\mathrm{PD}}^{-1}$ are Umklaap phonon–phonon, boundary and point defect scattering rates, respectively, defined as [5]:

(4) $$\tau_{U}^{-1}=\frac{k_{B}^{2}\gamma_{j}^{2}}{M\hbar v^{2}\theta_{D,j}}x^{2}T^{3}e^{-\frac{\theta_{D,j}}{3T}}$$

(5) $$\tau_{B}^{-1}=\frac{v}{d}$$

(6) $$\tau_{\mathrm{PD}}^{-1}=\frac{V_{0}\omega^{4}\Gamma }{4\pi v^{3}}$$

where *M* = 6.2231 × 10^−26^ kg/atom is average atomic mass, *γ_i_* is Grüneisen parameter, *d* is the sample thickness, *V*_0_ is the volume per atom, and *Γ* is the phonon mass-defect scattering parameter.

The Debye model has been widely used to describe phonon transport and size-dependent thermal conductivity in β-Ga_2_O_3_ [4,5,30]. The intrinsic anisotropy of β-Ga_2_O_3_ is accounted for by using direction-specific model parameters; in the present work, the parameters corresponding to the (−201) orientation were adopted [4,30] (see Table 1).

## 3. Results

### 3.1. Structural and Morphological Evolution of β-Ga_2_O_3_ Films with Thickness

**Figure 1:**
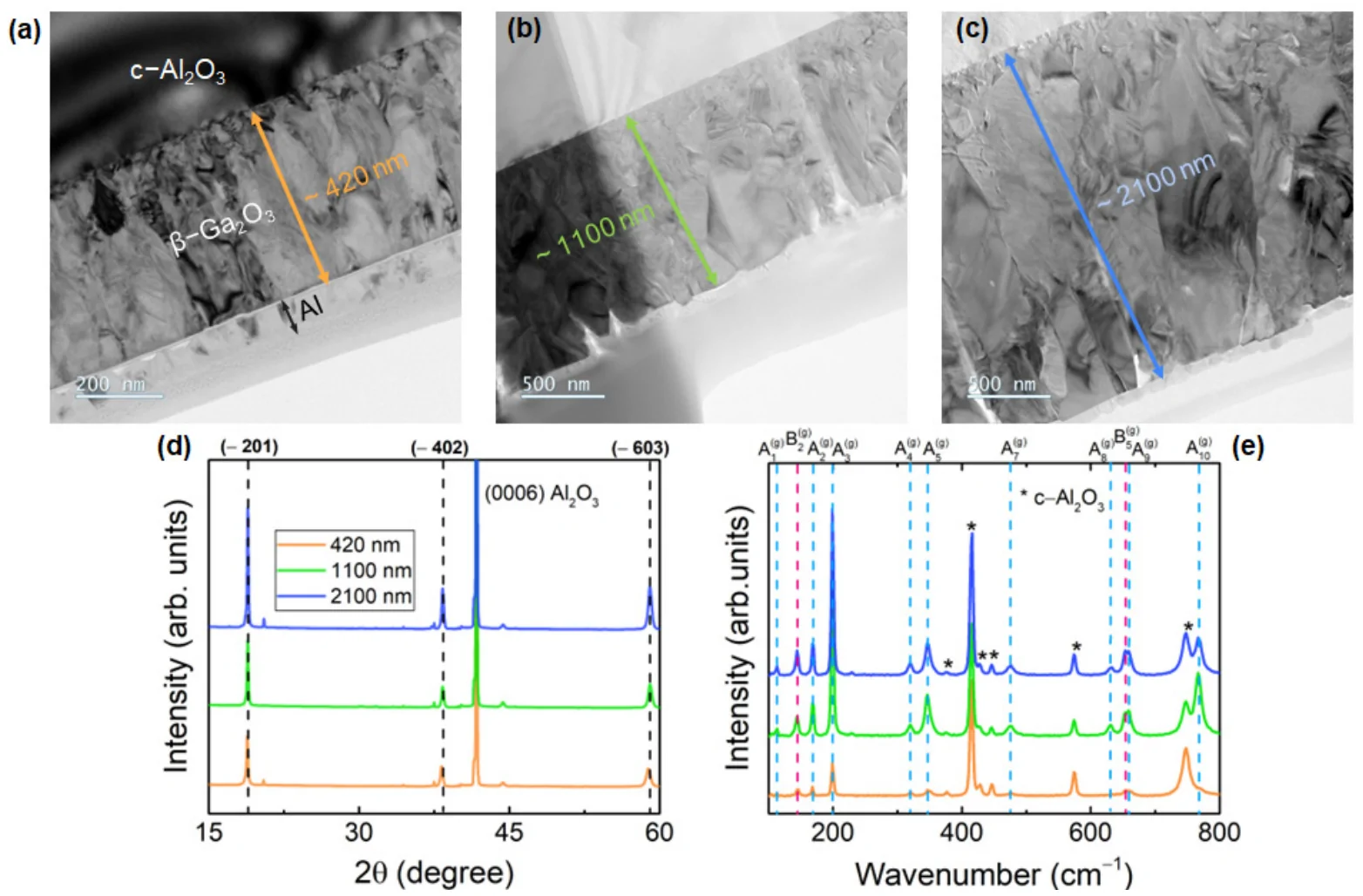
Structural characterization of RF-sputtered β-Ga_2_O_3_ films with different thicknesses. Cross-sectional bright-field transmission electron microscopy (BF-TEM) images of the (**a**) 420 nm, (**b**) 1100 nm, and (**c**) 2100 nm films. (**d**) X-ray diffraction (XRD) patterns of the β-Ga_2_O_3_ films with different thicknesses. Vertical dashed lines indicate the standard diffraction peak positions of monoclinic β-Ga_2_O_3_ corresponding to the (−201), (−402), and (−603) reflections according to JCPDS card No. 43-1012. (**e**) Raman spectra of the corresponding films. Blue and red dashed lines mark the characteristic A_g_ and B_g_ Raman modes of monoclinic β-Ga_2_O_3_, respectively, while asterisks denote Raman features originating from the sapphire substrate.

Figure 1 summarizes the structural characteristics of the β-Ga_2_O_3_ films with different thicknesses. Cross-sectional bright-field transmission electron microscopy (BF−TEM) images in Figure 1a–c reveal continuous β-Ga_2_O_3_ layers with thicknesses of approximately 420, 1100, and 2100 nm, respectively. All films exhibit a pronounced granular/columnar microstructure, while the characteristic dimensions of the crystalline domains evolve significantly with increasing film thickness. The 420 nm film consists of comparatively fine domains, whereas substantially larger crystalline regions are observed in 1100 and 2100 nm-thick films. Such thickness-dependent microstructural changes are qualitatively consistent with grain coarsening and competitive growth of crystalline domains, processes well documented during the growth of vapor-deposited thin films [31]. However, higher-magnification BF-TEM images of the thicker films reveal additional structural imperfections, such as bright cavity-like regions, void-like structures and intergranular porosity (see Figure S5, Supplementary Materials). The void-like and intergranular low-density regions observed in the thicker films may originate from incomplete coalescence of neighboring crystalline domains during competitive growth. As the film thickness increases, anisotropic growth and the development of faceted crystallites can produce a nonuniform growth front, leaving local gaps between adjacent domains that may become partially enclosed during continued deposition [31]. Although the high deposition temperature promotes surface diffusion, such local morphological irregularities may persist due to differences in facet-dependent growth rates and incomplete lateral coalescence. Similar morphological evolution involving faceting and growth-related heterogeneities has been reported in sputtered ZnO films [32], while void-related defects have also been observed in sputtered AlN films [33]. Nevertheless, some of the more diffuse BF-TEM contrast may also be attributed to a localized strain.

The surface morphology observed by SEM further supports this thickness-dependent microstructural evolution (see Figure S6, Supplementary Materials). The 420 nm film exhibits relatively fine surface features with characteristic lateral dimensions of approximately 100–200 nm. A pronounced coarsening is observed for the 1100 nm film, where elongated surface features extend from several hundred nanometers to approximately 1–2 μm. In contrast, the characteristic lateral dimensions of the 2100 nm film are somewhat smaller, typically on the order of several hundred nanometers. The evolution of the surface morphology is therefore non-monotonic, where substantial coarsening occurs between 420 and 1100 nm, followed by a moderate reduction in characteristic lateral feature size for the thickest film.

The XRD patterns of all three films exhibit characteristic reflections corresponding to the (−201), (−402), and (−603) crystallographic planes of monoclinic β-Ga_2_O_3_, indicating a pronounced (−201) preferred orientation as shown in Figure 1d. For the 420 nm film, these reflections are located at 2θ = 18.83°, 38.2°, and 58.84°, respectively. The corresponding peak positions are 18.88°, 38.31°, and 59° for the 1100 nm film, and 18.88°, 38.32°, and 58.99° for the 2100 nm film. The XRD results are also in agreement with previous reports on highly oriented β-Ga_2_O_3_ thin films grown by high-temperature sputtering and related epitaxial techniques [19,34] and the standard diffraction peak positions of monoclinic β-Ga_2_O_3_ according to JCPDS card No. 43-1012. No additional peaks related to other Ga_2_O_3_ polymorphs or secondary crystalline phases were detected within the sensitivity of the measurements geometry.

Raman spectroscopy measurements further confirmed the formation of monoclinic β-Ga_2_O_3_ in all investigated films, as shown in Figure 1e. The spectra of all three films exhibit the characteristic A_g_ and B_g_ modes of β-Ga_2_O_3_, which are in good agreement with the reported Raman response of monoclinic β-Ga_2_O_3_ [35]. The positions of the principal Raman bands remain essentially unchanged among the three films, indicating preservation of the β-phase over the investigated thickness range. In addition, the Raman peak intensity increases systematically with increasing film thickness due to the increased sampling volume, which is in agreement with the improved crystalline ordering and grain coarsening observed by electron microscopy and XRD analyses.

**Figure 2:**
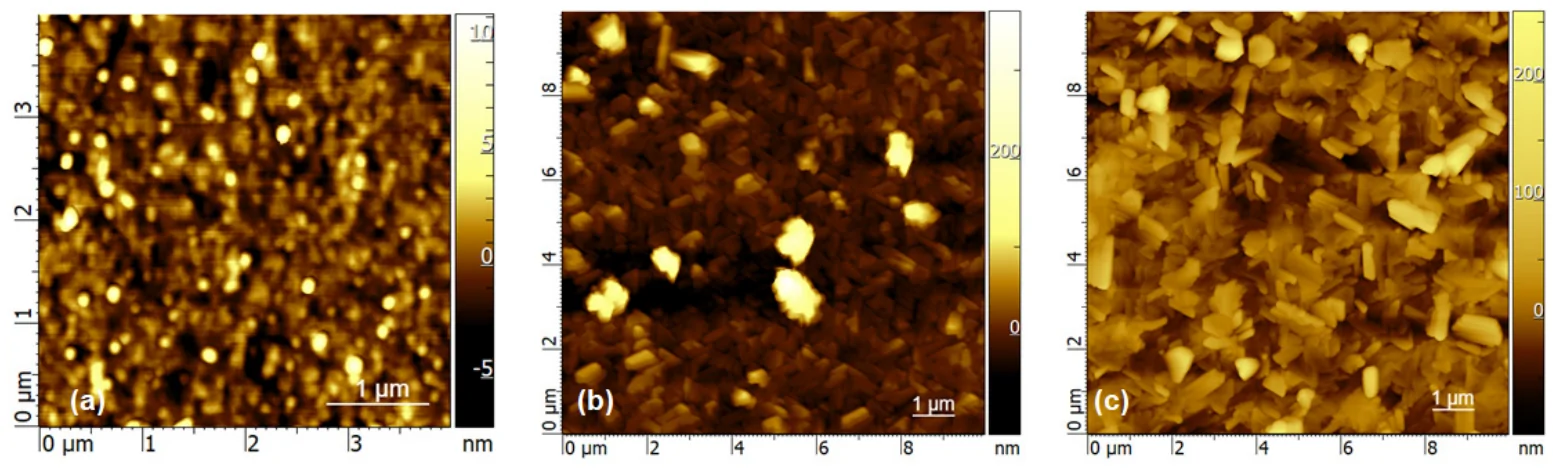
Surface morphology of RF-sputtered β-Ga_2_O_3_ films with thicknesses of (**a**) 420 nm, (**b**) 1100 nm, and (**c**) 2100 nm measured by AFM. For the 420 nm sample, a topographic map was acquired over an area of 4 × 4 μm^2^, whereas for the thicker samples, the maps were acquired over scan areas of 10 × 10 µm^2^.

The surface morphology of the β-Ga_2_O_3_ films was further examined by AFM over a scan areas of 4 × 4 µm^2^ for the 420 nm – thick sample and 10 × 10 μm^2^ for the thicker samples, as shown in Figure 2. The 420 nm film exhibits a relatively fine and uniform surface morphology with a surface roughness of approximately 1–2 nm. In contrast, the thicker films show a pronounced increase in surface topography, with roughness values in the range of approximately 20–30 nm. The 1100 nm film exhibits substantial lateral coarsening together with the formation of strongly faceted and elongated surface features, whereas the 2100 nm film retains a pronounced faceted morphology with a denser distribution of surface features. The AFM observations are consistent with the SEM results (see Supplementary Materials) and demonstrate a substantial evolution from a comparatively smooth, fine-featured surface in the thinnest film to a considerably rougher and more strongly faceted morphology in the thicker films. Together with the SEM and TEM observations, the AFM results indicate that film thickening is accompanied by pronounced domain coarsening and surface faceting, while the thicker films also exhibit void-like defects and intergranular porosity.

### 3.2. Thickness-Dependent Thermal Conductivity of β-Ga_2_O_3_ Films

**Figure 3:**
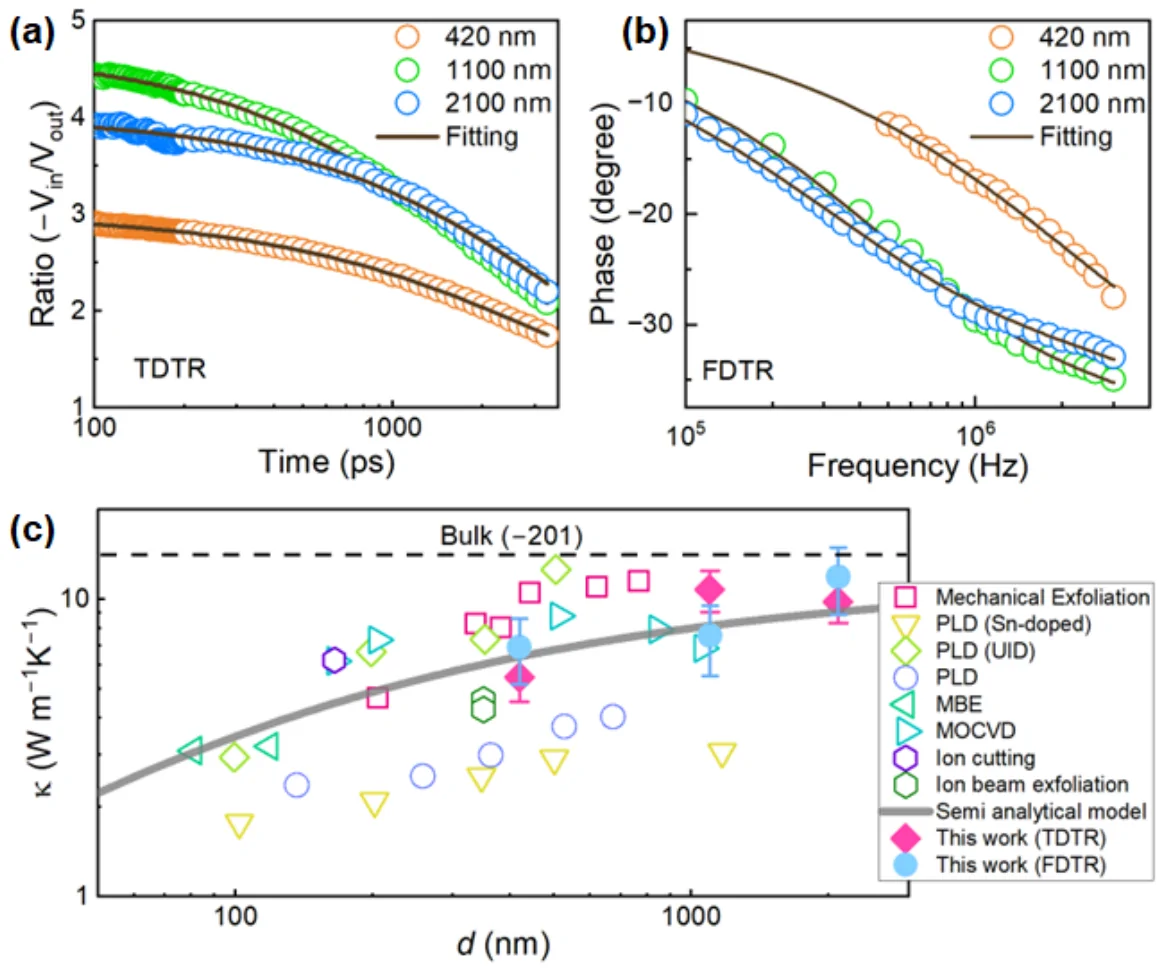
Thickness-dependent thermal transport in RF-sputtered β-Ga_2_O_3_ films. (**a**) Representative TDTR signals and (**b**) FDTR phase responses for films of different thicknesses. Open circles represent the experimental data, while black solid lines show the corresponding fits obtained using the multilayer heat-diffusion model. (**c**) Thermal conductivity as a function of β-Ga_2_O_3_ film thickness. Solid symbols represent the thermal conductivities obtained in the present work by TDTR and FDTR, whereas open symbols show literature values reported for β-Ga_2_O_3_ thin films fabricated using different approaches [11,12,13,14,24,36]. The solid line represents the analytical Debye-model calculation described by Equation (1), and the black dashed line indicates the literature bulk thermal conductivity of β-Ga_2_O_3_ for the (−201) orientation [4].

The thermal conductivity of the RF-sputtered films was independently evaluated using TDTR and FDTR. Representative experimental data together with the corresponding multilayer heat-diffusion model fits are shown in Figure 3a,b. The measured TDTR ratio (−V_in_/V_out_) is well reproduced by the model over the investigated pump–probe delay range, while the frequency-dependent FDTR phase response similarly shows good agreement with the calculated curves. The extracted thermal conductivity exhibits an overall increase with film thickness, although the dependence is not strictly monotonic, as shown in Figure 3c. The TDTR values rise from approximately 5.4 ± 1 W m^−1^ K^−1^ for the 420 nm film to 10.8 ± 1.7 W m^−1^ K^−1^ for the 1100 nm film, followed by a slight decrease to 9.8 ± 1.5 W m^−1^ K^−1^ for the 2100 nm film. This difference for thicker samples is smaller than the combined measurement uncertainties and therefore cannot be reliably resolved. In contrast, the FDTR-derived conductivity increases from approximately 6.9 ± 1.7 to 11.9 ± 3 W m^−1^ K^−1^ over the same thickness range. The uncertainty of the extracted thermal conductivity was assessed using a sensitivity analysis provided in the Supplementary Materials.

It is worth noting that the differences between the thermal conductivity values obtained by TDTR and FDTR may partly arise from the different heat-flow geometries and thermal length scales probed by the two techniques. In the present TDTR configuration, the relatively large laser spot size and high modulation frequency favor predominantly 1D cross-plane heat transport. In contrast, the FDTR measurements were performed using a smaller laser spot and lower modulation frequencies, under which lateral heat spreading becomes more pronounced. Consequently, the thermal conductivity extracted from FDTR can be regarded as an effective value reflecting contributions from both cross-plane and in-plane heat transport. The structural characterization suggests that microstructural heterogeneity may further contribute to the differences between the two techniques. In particular, BF-TEM images reveal void-like defects and defect-rich regions in the thicker films, while SEM and AFM show pronounced grain coarsening and increased surface roughness. Such spatial variations in defect density and grain structure can influence heat transport differently depending on the probed thermal depth and heat-flow direction.

Additionally, Figure 3c compares the present results with previously reported thermal conductivities of β-Ga_2_O_3_ thin films fabricated by different fabrication methods, including mechanical exfoliation [12], pulsed laser deposition (PLD) [11,36], molecular beam epitaxy (MBE) [13], ion cutting [14] and ion-beam exfoliation [24]. Despite the considerable scatter among the literature data, the thermal conductivity generally increases with increasing film thickness and gradually approaches the bulk value. The present TDTR and FDTR measurements fall within the range reported for β-Ga_2_O_3_ films fabricated by other techniques and follow the same overall thickness-dependent behavior.

The analytical phonon scattering model described in Equation (1) reproduces the overall thickness dependence of the measured thermal conductivity reasonably well. The increase in thermal conductivity with film thickness is primarily associated with the reduced influence of phonon-boundary scattering. The point-defect scattering parameter obtained from the best-fit of the model is *Γ* = 4 × 10^–13^, indicates that point defect scattering plays only a minor role compared with Umklapp and boundary scattering. It should be emphasized, however, that this fitted scattering parameter does not explicitly account for extended microstructural defects, such as grain boundaries, dislocations, or the void-like/intergranular defects observed by TEM but reflects an effective contribution within the model. The deviations between the calculated and measured thermal conductivities may therefore partly originate from microstructural features that are not explicitly represented in the simple analytical model. Advanced approaches, such as molecular dynamics simulations using machine-learning interatomic potentials [37,38], can be used to capture the effects of complex and extended defects. Nevertheless, the experimentally observed increase in thermal conductivity with thickness indicates that the reduction in finite-size/boundary scattering remains the dominant trend across the investigated thickness range.

### 3.3. Effect of Ar Plasma Treatment on Al/β-Ga_2_O_3_ Interfacial Thermal Transport

**Figure 4:**
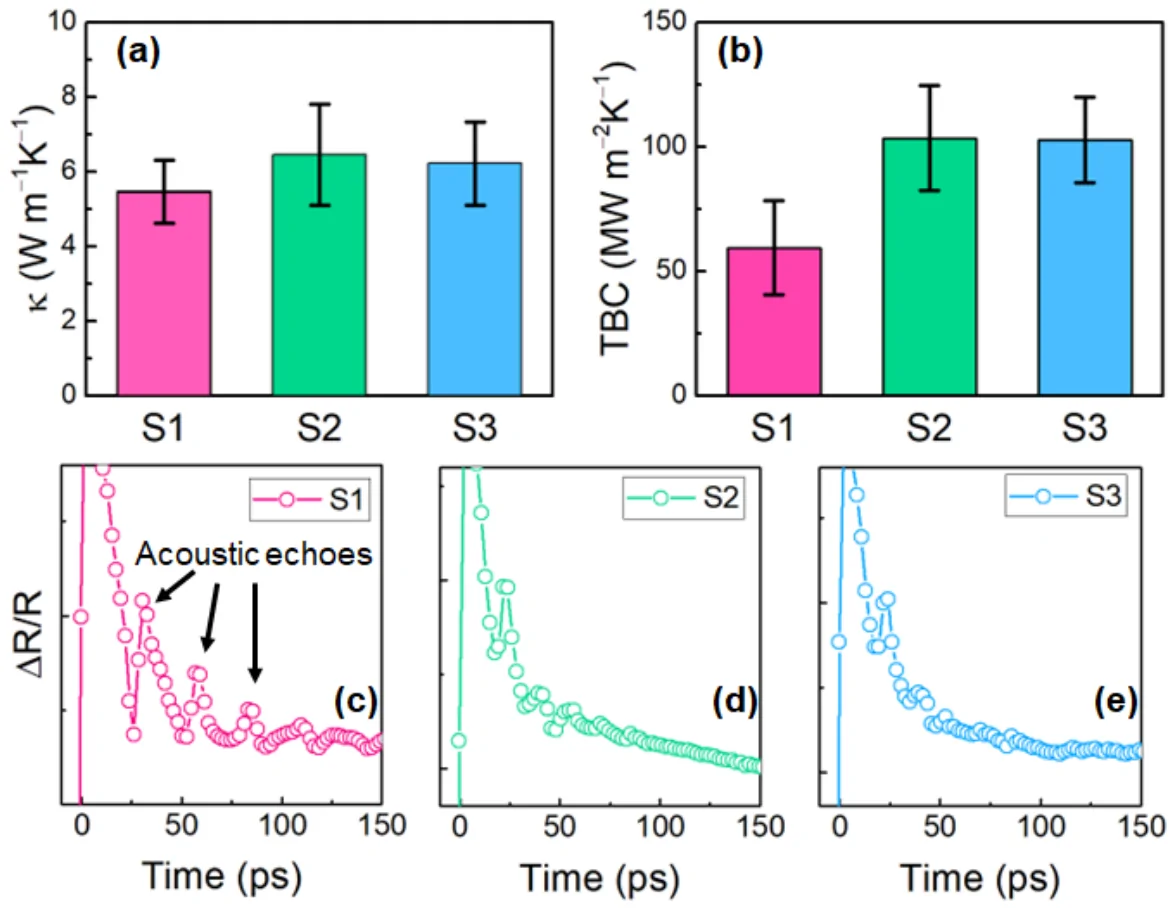
Effect of Ar plasma surface treatment on thermal transport across the Al/β-Ga_2_O_3_ interface. (**a**) Thermal conductivity of the β-Ga_2_O_3_ films and (**b**) thermal boundary conductance (TBC) of the Al/β-Ga_2_O_3_ interface for samples S1–S3. S1 corresponds to the untreated sample, whereas S2 and S3 were exposed to Ar plasma at 50 W for 5 min and 100 W for 2 min, respectively. (**c**–**e**) Corresponding picosecond acoustic signals measured for (**c**) S1, (**d**) S2, and (**e**) S3.

To investigate the effect of Ar plasma surface treatment on thermal transport across the Al/β-Ga_2_O_3_ interface, the thermal conductivity and TBC were measured for samples S1–S3, as shown in Figure 4. Sample S1 was left untreated, whereas S2 and S3 were exposed to Ar plasma at 50 W for 5 min and 100 W for 2 min, respectively. The measured β-Ga_2_O_3_ thermal conductivity varies only moderately among the three samples, remaining within approximately 5–7 W m^−1^ K^−1^. A pronounced change is observed in the Al/β-Ga_2_O_3_ TBC, as shown in Figure 4b. The plasma-treated samples exhibit TBC values of approximately 100 MW m^−2^ K^−1^, about 70% higher than the untreated sample (~60 MW m^−2^ K^−1^). Picosecond acoustic measurements provide complementary information on the interfacial response as shown in Figure 4c–e. The untreated S1 sample exhibits a pronounced sequence of coherent acoustic echoes following the initial response of the Al transducer, indicating repeated reflection of the acoustic strain pulse within the Al layer. In contrast, the higher-order echoes are strongly suppressed for the plasma-treated S2 and S3 samples, for which the acoustic response decays more rapidly after the first few oscillations. This clear difference demonstrates that Ar plasma treatment substantially modifies the acoustic response of the Al/β-Ga_2_O_3_ interface.

A qualitatively similar behavior was reported by Zheng et al., who observed considerably faster damping of acoustic echoes at Al/nonmetal interfaces exhibiting higher TBC and established a correlation between thermal conductance and acoustic transmission coefficient [22]. They attributed the enhancement primarily to stronger interfacial coupling resulting from surface-contamination removal and the formation of stronger interfacial bonds during surface treatment or sputter deposition. In their study, plasma pretreatment also increased the TBC of Al/Al_2_O_3_, Al/GaN, and Al/Si interfaces, supporting the general concept that modification of the nonmetal surface prior to Al deposition can substantially alter interfacial phonon transport.

**Figure 5:**
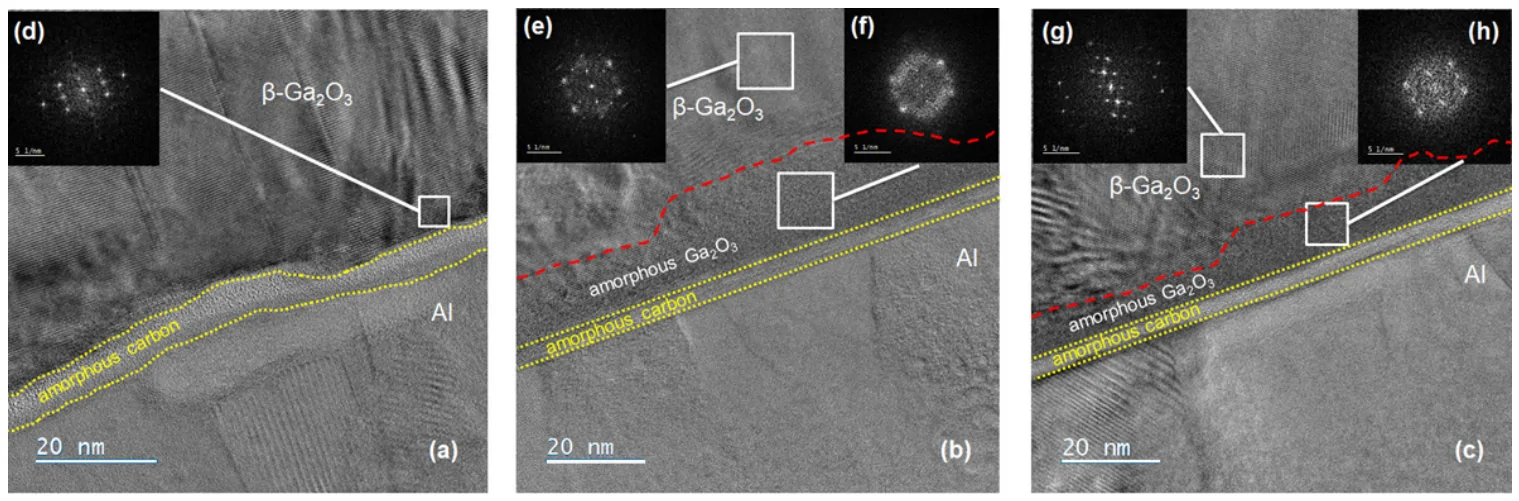
Cross-sectional TEM characterization of the Al/β-Ga_2_O_3_ interfaces without and with Ar plasma treatment. High-resolution BF-TEM images of (**a**) untreated S1, (**b**) S2 treated at 50 W for 5 min, and (**c**) S3 treated at 100 W for 2 min. (**d**–**h**) Fast Fourier transform (FFT)patterns acquired from the regions indicated by the white squares in panels (**a**–**c**). Yellow dotted lines mark the amorphous carbon-containing surface-contamination layer observed at the interfaces, whereas the red dashed lines in panels (**b**,**c**) define the plasma-induced amorphous/disordered Ga_2_O_3_ region. The corresponding SAED patterns confirm the structural differences between the crystalline β-Ga_2_O_3_ film and the disordered interfacial regions.

To clarify the structural origin of the plasma-induced change in TBC, the Al/β-Ga_2_O_3_ interfaces of S1–S3 were examined by cross-sectional HRTEM, as shown in Figure 5. The untreated S1 sample exhibits a comparatively abrupt transition between the crystalline β-Ga_2_O_3_ film and the interfacial region beneath the Al transducer. A thin carbon-containing layer is also present at the interface in all three samples, most likely originating from surface contamination prior to Al deposition. Since this layer is observed in both untreated and plasma-treated samples, its presence alone cannot account for the substantial difference in TBC. In contrast, both plasma-treated samples, S2 and S3, exhibit a thin partially amorphized region at the β-Ga_2_O_3_ surface directly beneath the Al layer. The corresponding FFTs from the indicated regions confirm that the underlying Ga_2_O_3_ film retains its crystalline β-phase after plasma treatment. Thus, the structural modification appears to be confined primarily to a thin near-surface region rather than extending throughout the film. This region is therefore attributed to near-surface structural modification induced by Ar plasma exposure.

**Figure 6:**
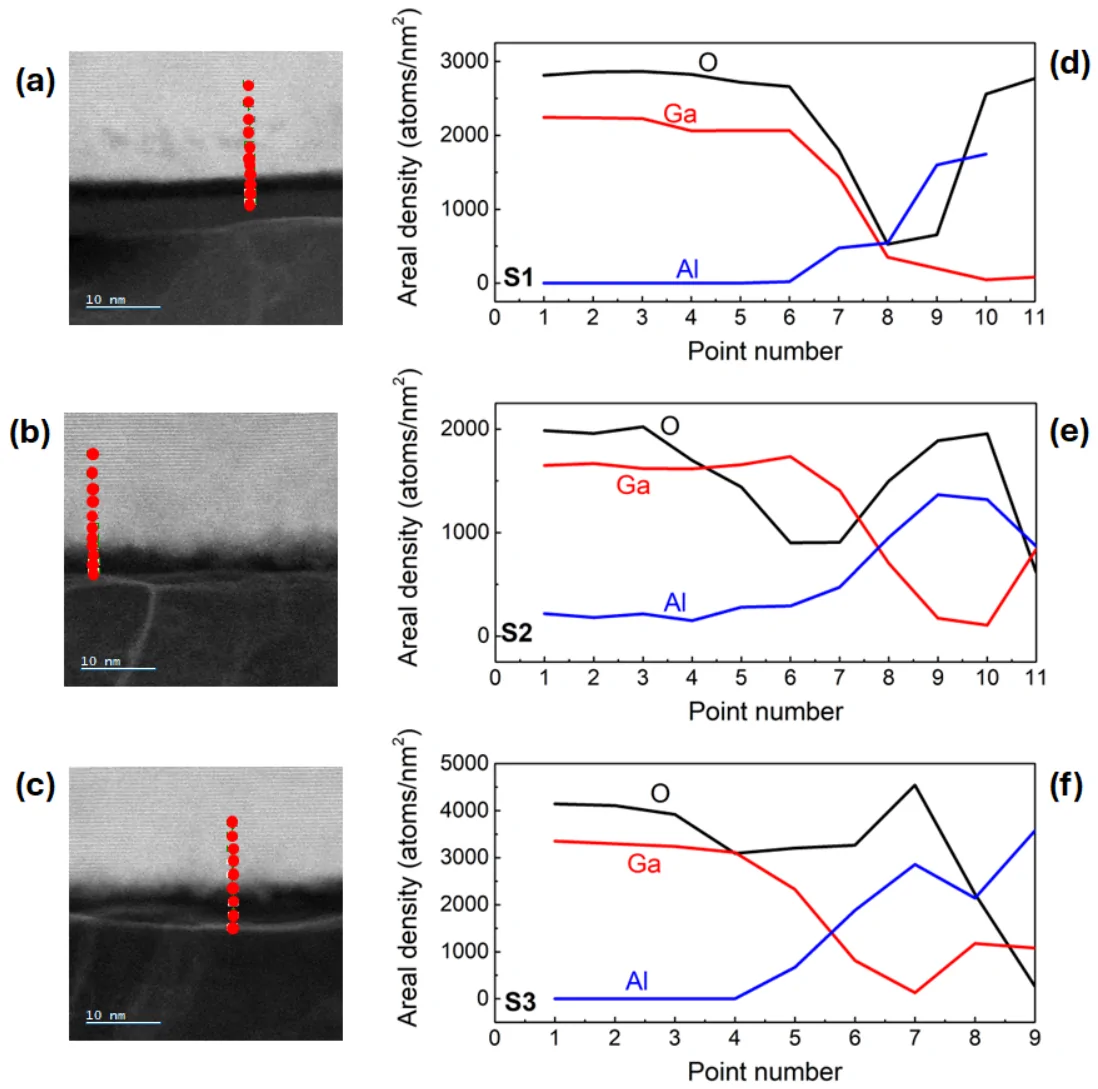
High-Angle Annular Dark-Field Scanning Transmission Electron Microscopy (HAADF-STEM) images (**a**,**b**,**c**) and corresponding EELS elemental areal-density profiles (**d**,**e**,**f**) of O, Ga, and Al across the Al/β-Ga_2_O_3_ interfaces for (**a**,**d**) untreated S1, (**b**,**e**) Ar plasma-treated S2 (50 W, 5 min), and (**c**,**f**) Ar plasma-treated S3 (100 W, 2 min). Red markers in the HAADF-STEM images indicate the EELS acquisition positions, with the corresponding point numbers shown on the horizontal axes of the elemental profiles. The Ga_2_O_3_ and Al layers are located at the top and bottom of the images, respectively.

To further examine the chemical modification of the near-interface region, spatially resolved EELS measurements were performed across the Al/β-Ga_2_O_3_ interfaces of S1, S2, and S3. As shown in Figure 6, the untreated S1 sample exhibits a drop in the O and Ga signals together at the edge of the Ga_2_O_3_ layer, indicating that no appreciable oxygen loss is observed prior to the interface. In contrast, for the plasma-treated samples S2 and S3, the O signal begins to decrease well before the interface is reached, whereas the Ga signal remains relatively high over the same region. Since the Ga profile serves as a reference for the presence of the Ga_2_O_3_ phase, this relative reduction of oxygen with respect to gallium indicates the formation of an oxygen-deficient near-surface region in the plasma-treated samples. The effect is particularly pronounced in S2. These EELS results are consistent with the HRTEM observations of a partially amorphized/disordered interfacial region and provide additional evidence that Ar plasma treatment modifies both the structure and local chemistry of the β-Ga_2_O_3_ surface prior to Al deposition. Nevertheless, while the data support oxygen deficiency in the near-interface region, they do not by themselves establish the exact oxygen-vacancy concentration or the detailed microscopic mechanism by which the modified interface enhances TBC.

## 4. Discussion

The target-racetrack treatment configuration suggests that direct ion bombardment and sputtering played an important role in modifying the β-Ga_2_O_3_ surface. However, since the uncovered parts of the Ga_2_O_3_ target continued to be sputtered, some redeposition of target-derived species cannot be excluded. The relatively low working pressure and coplanar arrangement of the sample with respect to the target surface are expected to limit direct redeposition, although gas-phase scattering may still contribute. In addition, ion bombardment may induce near-surface structural disorder and possible stoichiometric changes. The ultrathin amorphous/disordered region observed by TEM is consistent with such ion-induced modification, but the available experimental data does not allow the individual contributions of sputtering, redeposition, and compositional changes to be quantitatively separated.

The formation of a thin partially amorphous Ga_2_O_3_ layer at the Al/β-Ga_2_O_3_ interface in S2 and S3, while the underlying film remains crystalline, demonstrates that Ar plasma treatment modifies the near-surface region governing interfacial heat transfer. This suggests that the enhancement may involve not only changes in interfacial bonding, but also the formation of an ultrathin transition region between Al and crystalline β-Ga_2_O_3_. Such a region may provide additional vibrational states that facilitate energy transfer across the interface by partially bridging the vibrational mismatch between the adjoining materials [39,40,41,42,43,44]. Several studies involving β-Ga_2_O_3_ support this possibility. Molecular dynamics simulations have shown that introducing a nanoscale SiO_2_ interlayer between β-Ga_2_O_3_ and SiC can increase the TBC by up to 3.7 times, with the enhancement attributed to improved phonon spectral overlap, particularly in the low-frequency range, and an increased phonon participation ratio [39]. Similarly, in our previous calculations for β-Ga_2_O_3_/Si, we demonstrated that an ultrathin amorphous SiO_2_ layer can act as a bridge between the vibrational modes of the two materials, with localized vibrational modes in the amorphous layer opening additional channels for thermal transport [24]. A related mechanism has recently been predicted for β-Ga_2_O_3_/Au interfaces, where oxygen-vacancy-induced disorder and interfacial mixing generate an alloy-like transition region that reduces the phonon mismatch and enhances interfacial heat transport [40]. Phonon-density-of-states analysis further showed enhanced overlap of low- and intermediate-frequency vibrational modes near the defective interface, providing additional pathways for phonon transmission [40]. More generally, theoretical studies of β-Ga_2_O_3_ interfaces containing two-dimensional interlayers have similarly shown that additional interfacial phonon modes can bridge the mismatch between the phonon spectra of adjoining materials and thereby increase TBC [41].

However, it should be emphasized that the presence of an amorphous or chemically modified interlayer does not universally enhance interfacial heat transport. Its effect depends strongly on the interlayer thickness, composition, bonding configuration, and vibrational properties. For example, in Ti/β-Ga_2_O_3_ interfaces, defective Ga_2_O_3_ together with a TiO_x_ reaction layer was shown to introduce additional thermal resistance, with the low thermal conductivity of the oxide layer reducing the overall TBC [20]. Similarly, reaction-generated oxide layers at metal/β-Ga_2_O_3_ interfaces have been reported to lower TBC as their thickness increases [21]. These results demonstrate that a structurally disordered or chemically modified interfacial region can either facilitate or impede heat transfer depending on its specific characteristics. In the present samples, the higher TBC values observed in S2 and S3 may be associated with the ultrathin disordered interfacial region, which could influence interfacial coupling and phonon transmission. However, because the samples were prepared in separate deposition runs, possible contributions from variations in the initial film and surface properties cannot be excluded. Therefore, the present results suggest a potential role of plasma-induced near-surface modification in controlling interfacial thermal transport.

Nevertheless, it is worth noting that the present study lacks of atomistic insight into the mechanisms governing thermal transport across the modified Al/β-Ga_2_O_3_ interfaces. Future investigations combining nonequilibrium molecular dynamics simulations with machine-learned interatomic potentials could clarify the influence of interfacial disorder, chemical bonding, and defect configurations on phonon transmission and identify interface structures with potentially higher thermal boundary conductance. Recent advances in machine-learning-based simulations of β-Ga_2_O_3_ heterointerfaces demonstrate the feasibility of such an approach [45,46]. Furthermore, systematic comparisons of different surface modification techniques, including alternative plasma treatments and surface activation methods, could provide additional routes for controlling interfacial heat transport. Complementary chemical characterization would help identify changes in local bonding, stoichiometry, and possible interfacial reactions, providing a more comprehensive understanding of the relationship between surface chemistry and thermal boundary conductance.

The thermal boundary conductance values reported for metal/β-Ga_2_O_3_ interfaces are summarized in Table 2, together with the results of the present study. Previous investigations have demonstrated substantial variations in TBC depending on the metal contact, deposition conditions, and interfacial structure. For example, Shi et al. reported experimental TBC values of 17.4–82.7 MW m^−2^ K^−1^ for Au, Ti, Ni, and Al contacts, whereas their ideal-interface calculations predicted values of approximately 71–149 MW m^−2^ K^−1^ [20]. Aller et al. demonstrated substantially higher effective TBC values through the introduction of ultrathin metallic interlayers, reaching approximately 530 MW m^−2^ K^−1^ for an Au/Cr/β-Ga_2_O_3_ structure [21]. More recently, Al/β-Ga_2_O_3_ interfaces prepared using ion-beam-exfoliated nanomembranes exhibited TBC values of approximately 40–70 MW m^−2^ K^−1^ [24]. In the present study, the Ar plasma-treated samples exhibit TBC values of approximately 100 MW m^−2^ K^−1^, compared with approximately 60 MW m^−2^ K^−1^ for the untreated sample. Despite these advances, experimental investigations of TBC at metal/β-Ga_2_O_3_ interfaces remain relatively limited, particularly regarding the effects of surface modification and interfacial structural disorder. Further systematic studies are therefore needed to develop effective strategies for improving interfacial heat transport in β-Ga_2_O_3_-based electronic devices.

## 5. Conclusions

In this work, we investigated thermal transport in high-temperature RF-sputtered β-Ga_2_O_3_ thin films by combining advanced thermoreflectance techniques with structural, morphological, and microscopic characterization. The films exhibit a pronounced thickness-dependent thermal conductivity, increasing from approximately 5–7 W m^−1^ K^−1^ at 420 nm to about 10–12 W m^−1^ K^−1^ for thicker films. This evolution is accompanied by substantial micro-structural changes, including grain/domain coarsening and the development of intergranular void-like defects. The overall thickness dependence is reasonably described by a Debye–Klemens model incorporating boundary and defect-related phonon scattering, highlighting the important role of microstructure in governing heat transport in sputtered β-Ga_2_O_3_.

The analytical framework therefore provides an efficient means of describing the thickness dependence observed here, while also clarifying the limits of its predictive capacity. Addressing these limits will require more advanced approaches, such as molecular dynamics simulations with machine-learning interatomic potentials, which can improve theoretical understanding of how complex defects affect both bulk thermal conductivity and thermal boundary conductance.

We further demonstrate that thermal transport at the Al/β-Ga_2_O_3_ interface can be modified independently through Ar plasma treatment. The plasma-treated samples exhibit TBC values of approximately 100 MW m^−2^ K^−1^, compared with approximately 60 MW m^−2^ K^−1^ in the untreated sample, corresponding to an enhancement of about 70%, while producing only a comparatively small change in the thermal conductivity of the underlying β-Ga_2_O_3_ film. The enhanced interfacial heat transfer is accompanied by a pronounced modification of the picosecond acoustic response and the formation of an ultrathin amorphous/disordered near-surface region, while the underlying β-Ga_2_O_3_ remains crystalline. Additional HAADF-EELS measurements reveal a relative decrease in the oxygen signal with respect to gallium near the interface in the plasma-treated samples, suggesting local oxygen deficiency within the modified region. Although possible variations between independent deposition runs cannot be entirely excluded, these observations indicate that plasma-induced modification of the near-surface structure and interfacial coupling plays an important role in the enhanced thermal boundary conductance.

Overall, the results demonstrate two complementary pathways for thermal-transport engineering in sputtered β-Ga_2_O_3_: film-scale control through thickness-dependent microstructural evolution and interface-scale control through plasma-induced surface modification. This provides a practical framework for tailoring both heat conduction within β-Ga_2_O_3_ layers and heat transfer across metal/β-Ga_2_O_3_ interfaces, with potential relevance to thermal management in β-Ga_2_O_3_-based power electronic devices.

## Figures and Tables

**Table 1:** Analytical model parameters for (−201) β-Ga_2_O_3_.

Phonon Branch	*θ_D_*_,*j*_, K [4]	*γ_i_* [30]
LA	183	1
TA1	90	2
TA2	90	2

**Table 2:** The thermal boundary conductance values reported for metal/β-Ga_2_O_3_ interfaces including experimental methods and modeling.

Metal/β-Ga_2_O_3_ Structure	Preparation/Modification	Interface Characteristics	Method	TBC (MW m^−2^ K^−1^)	Reference
Au/β-Ga_2_O_3_	Wedge deposition	No intentional interlayer	FDTR	45	[21]
Au/β-Ga_2_O_3_	E-beam evaporation	Defective interfacial region	TDTR	31.2	[20]
Ti/β-Ga_2_O_3_	E-beam evaporation	Defective/reaction region	TDTR	17.4	[20]
Ni/β-Ga_2_O_3_	E-beam evaporation	Defective interfacial region	TDTR	82.7	[20]
Al/β-Ga_2_O_3_	E-beam evaporation	Defective interfacial region	TDTR	81.7	[20]
Au/Cr/β-Ga_2_O_3_	Wedge deposition	2.5 nm Cr interlayer	FDTR	530	[21]
Au/Ti/β-Ga_2_O_3_	Wedge deposition	5 nm Ti interlayer	FDTR	260	[21]
Au/Ni/β-Ga_2_O_3_	Wedge deposition	Ni interlayer (≥3 nm)	FDTR	410	[21]
Al/β-Ga_2_O_3_	Al on mechanically exfoliated films	No intentional interlayer	TDTR	30–150	[12]
Al/β-Ga_2_O_3_	Ion-beam-exfoliated nanomembranes	Sputtered Al, no dedicated surface treatment	TDTR	40–70	[24]
Al/β-Ga_2_O_3_	RF-sputtered β-Ga_2_O_3_, untreated S1	Untreated reference	TDTR	~59.6	This work (S1)
Al/β-Ga_2_O_3_	RF-sputtered β-Ga_2_O_3_, Ar plasma 50 W, 5 min	Amorphous/disordered interfacial region	TDTR	~103.6	This work (S2)
Al/β-Ga_2_O_3_	RF-sputtered β-Ga_2_O_3_, Ar plasma 100 W, 2 min	Amorphous/disordered interfacial region	TDTR	~102.9	This work (S3)
Au/β-Ga_2_O_3_	Ideal-interface modeling	No interlayer	DMM	71.2	[20]
Ti/β-Ga_2_O_3_	Ideal-interface modeling	No interlayer	DMM	103.0	[20]
Ni/β-Ga_2_O_3_	Ideal-interface modeling	No interlayer	DMM	126.0	[20]
Al/β-Ga_2_O_3_	Ideal-interface modeling	No interlayer	DMM	139.6	[20]

## Data Availability

The original contributions presented in this study are included in the article/supplementary material. Further inquiries can be directed to the corresponding authors.

## Supplementary Materials

The following supporting information can be downloaded at: https://www.mdpi.com/article/10.3390/nano16211439/s1, The Supplementary Materials include additional SEM and TEM characterization of the β-Ga_2_O_3_ films, as well as sensitivity analyses for the TDTR and FDTR measurements. References [12,23,26,27,28,47,48,49] are cited in the supplementary materials.

## Abbreviations

The following abbreviations are used in this manuscript:

UWBG	Ultrawide-bandgap
β-Ga_2_O_3_	Beta gallium oxide
RF	Radio-frequency
TBC	Thermal boundary conductance
XRD	X-ray diffraction
AFM	Atomic force microscopy
HRTEM	High-resolution transmission electron microscopy
HAADF-STEM	High-angle annular dark-field scanning transmission electron microscopy
SEM	Scanning electron microscopy
TDTR	Time-domain thermoreflectance
FDTR	Frequency-domain thermoreflectance
V_in_/V_out_	Ratio of the in-phase and out-of-phase lock-in signals
$\theta_{D,j}$	Debye temperature
τ	Total phonon relaxation time
T	Temperature
v	Average phonon velocity
*C(x)*	Lattice specific heat
*k_B_*	Boltzmann’s constant
ℏ	Reduced Planck’s constant
LA	Longitudinal acoustic
TA	Transverse acoustics
$\tau_{U}^{-1}$	Umklaap phonon–phonon scattering rates
$\tau_{B}^{-1}$	Boundary scattering rates
$\tau_{\mathrm{PD}}^{-1}$	Point defect scattering rates
M	Average atomic mass,
$\gamma_{i}$	Grüneisen parameter
d	Sample thickness
$V_{0}$	Volume per atom
Γ	Phonon scattering cross section.
BF-TEM	Bright-field transmission electron microscopy
A_g_ and B_g_	Characteristic modes of beta gallium oxide
PLD	Pulsed laser deposition
MBE	Molecular beam epitaxy
FFT	Fast Fourier Transform
EELS	Electron energy loss spectroscopy
