# Supplementary material for: Tuning Thermal Transport in Radio-Frequency-Sputtered Beta-Gallium Oxide Through Thickness and Plasma-Induced Interface Modification
Source: Nanomaterials (Basel). 2026 Nov 6;16(21):1439. doi: 10.3390/nano16211439 (PMC13764965; doi:10.3390/nano16211439)
Supplement: Supplementary file 1 [file nanomaterials-16-01439-s001.zip › nanomaterials-4625568-supplementary.pdf]

Supplementary Materials

Tuning Thermal Transport in Radio-Frequency-Sputtered Beta-Gallium Oxide Through Thickness and Plasma-Induced Interface Modification

Azat Abdullaev[1,2,3*], Rustem Tlegenov[1,2,3], Magomed A. Umakhanov[4], Jacques O'Connell[5], Abubakar M. Ismailov[4], Kairolla Sekerbayev[1,2,3], Zhandos N. Utegulov[2*]

[1]Center for Energy and Advanced Materials Science, National Laboratory Astana, Astana 010000, Kazakhstan
[2]Department of Physics, School of Sciences and Humanities, Nazarbayev University, Astana 010000, Kazakhstan
[3]Institute of New Materials and Energy Technologies, 53 Kabanbay Batyr Ave., 010000 Astana, Kazakhstan
[4]Dagestan State University, Makhachkala, Republic of Dagestan, Russia
[5]Centre for HRTEM, Nelson Mandela University, Port Elizabeth, 6001, South Africa

Corresponding authors: azat.abdullaev@nu.edu.kz; zhutegulov@nu.edu.kz.

The sensitivity of the TDTR and FDTR was evaluated to assess the reliability of the extracted thermal properties. For TDTR, the sensitivity coefficient for a parameter $p_i$was defined as

$$S_i^{\mathrm{TDTR}} = \frac{\partial \ln(-V_{\mathrm{in}}/V_{\mathrm{out}})}{\partial \ln p_i},$$

where $-V_{\mathrm{in}}/V_{\mathrm{out}}$is the TDTR ratio signal [23, 27]. For FDTR, the sensitivity was evaluated from the variation of the thermoreflectance phase $\theta$with respect to the model parameter,

$$S_i^{\mathrm{FDTR}} = \frac{\partial \theta}{\partial \ln p_i},$$

following the conventional FDTR analysis [28]. A larger absolute sensitivity indicates a stronger dependence of the measured signal on the corresponding parameter.

The TDTR sensitivity coefficients for the 420, 1100, and 2100 nm β-Ga_2O_3 films are shown in Fig. S1. For all three samples, the TDTR signal exhibits appreciable sensitivity to the cross-plane thermal conductivity, κ_z, of the β-Ga_2O_3 layer over the delay-time range used for fitting due to a large beam spot size and low heat penetration depth which is expressed as ($D_{th} = \sqrt{\frac{\kappa}{\pi C_v f}}$), where κ – thermal conductivity, C_v – volumetric specific heat capacity and f – modulation frequency. In contrast, the sensitivity to the in-plane thermal conductivity, κ_r, remains close to zero, confirming that the combination of the relatively large laser spot and the MHz modulation

frequency predominantly probes cross-plane heat transport. The sensitivity to the sapphire thermal conductivity is negligible under these conditions. The signal shows substantial sensitivity to the β-Ga_2O_3 volumetric heat capacity and particularly strong sensitivity to the Al transducer thickness. This behavior is consistent with previous TDTR studies, in which the transducer thickness and beam dimensions represent important sources of uncertainty in thin-film thermal conductivity measurements [12, 23]. The sensitivity to the Al/β-Ga_2O_3 TBC is also given in the figure and exhibits a delay-time dependence.

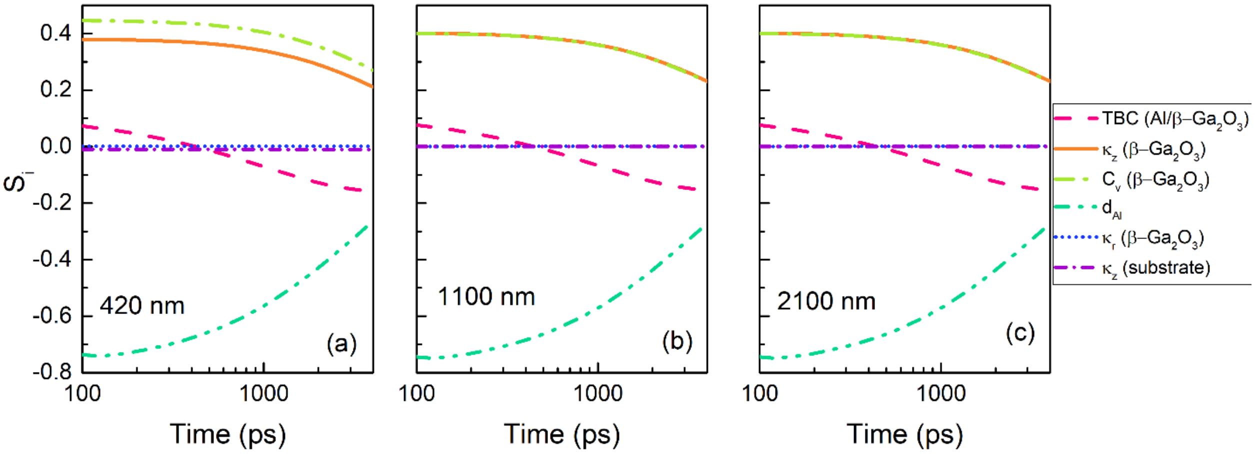

Figure S1. TDTR sensitivity coefficients for the (a) 420 nm, (b) 1100 nm, and (c) 2100 nm β-Ga_2O_3 films as a function of pump–probe delay time. The sensitivities are shown for the Al/β-Ga_2O_3 thermal boundary conductance (TBC), cross-plane (κ_z) and in-plane (κ_x) thermal conductivities of β-Ga_2O_3, β-Ga_2O_3 volumetric heat capacity (C_v), Al transducer thickness, and sapphire substrate thermal conductivity.

The corresponding FDTR sensitivity coefficients are shown in Fig. S2. The FDTR phase exhibits strong frequency−dependent sensitivity to the effective thermal conductivity, κ_{eff}, of the β-Ga_2O_3 films, with the magnitude increasing toward the higher−frequency region. The strongest sensitivity is observed for the effective pump–probe beam radius, R_0, demonstrating the importance of an accurate determination of the laser spot size for FDTR analysis. This behavior results from the pronounced three-dimensional heat spreading associated with the smaller beam size used in FDTR measurements and is consistent with previous FDTR studies [26, 28]. The phase also shows appreciable sensitivity to the Al/β−Ga_2O_3 TBC, particularly at higher modulation frequencies. In contrast, the sensitivity to the buried β−Ga_2O_3/sapphire interface decreases strongly with increasing film thickness. A measurable sensitivity remains for the 420 nm film, whereas it becomes considerably smaller for the 1100 nm film and nearly negligible for the 2100 nm film.

The thermophysical properties used as fixed inputs in the multilayer heat-diffusion model were taken from the literature, including the volumetric heat capacities of Al, β−Ga_2O_3, and sapphire and the thermal conductivities of the Al transducer and sapphire substrate [47–49].

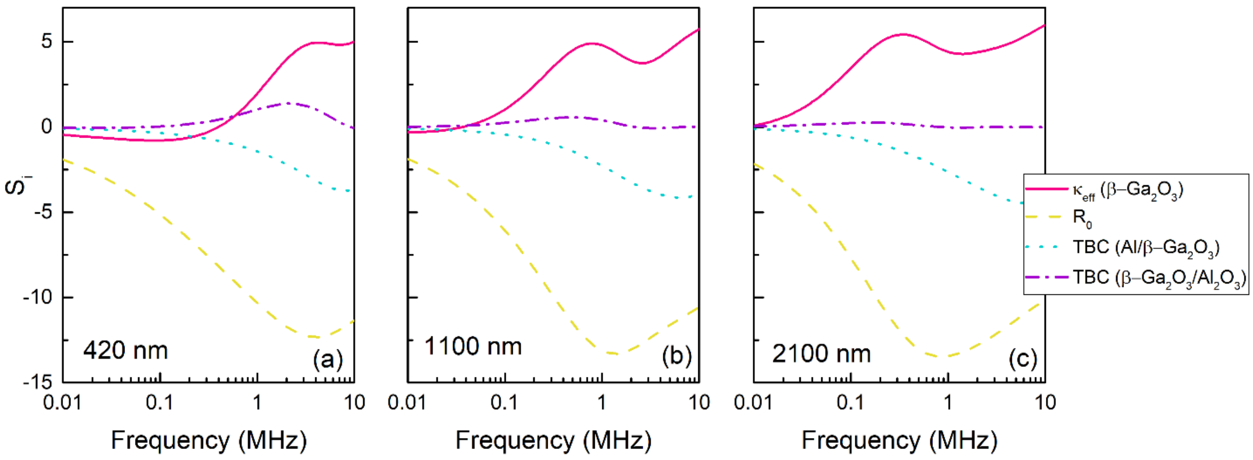

Figure S2. FDTR sensitivity coefficients for the (a) 420 nm, (b) 1100 nm, and (c) 2100 nm β-Ga_2O_3 films as a function of pump modulation frequency. The sensitivities are shown for the effective β−Ga_2O_3 thermal conductivity, effective pump–probe beam radius R_0, Al/β−Ga_2O_3 TBC, and β−Ga_2O_3/sapphire TBC.

To evaluate the spatial reproducibility of the thermal measurements, TDTR and FDTR measurements were performed at 4−5 spatially separated locations on each sample. Figure S3 shows the individual thermal conductivity values obtained for β-Ga_2O_3 films with thicknesses of 420, 1100, and 2100 nm. Figure S4 shows the spatial variation in the thermal conductivity and Al/β-Ga_2O_3 TBC for the untreated (S1) and Ar plasma-treated (S2 and S3) samples. The error bars are associated with individual measurements uncertainties arising from the fitting procedure and variations in the fixed parameters of the multilayer heat-diffusion model.

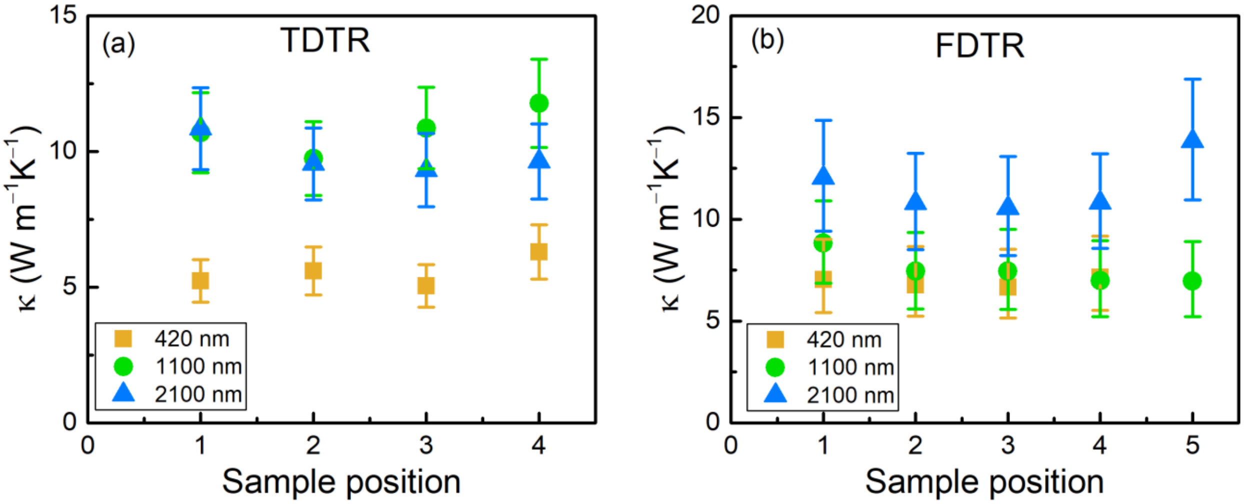

Figure S3. Spatial variation in the thermal conductivity (κ) of β-Ga_2O_3 thin films with thicknesses of 420, 1100, and 2100 nm, measured using (a) TDTR and (b) FDTR at different locations on each sample. Error bars represent the fitting uncertainties of individual measurements.

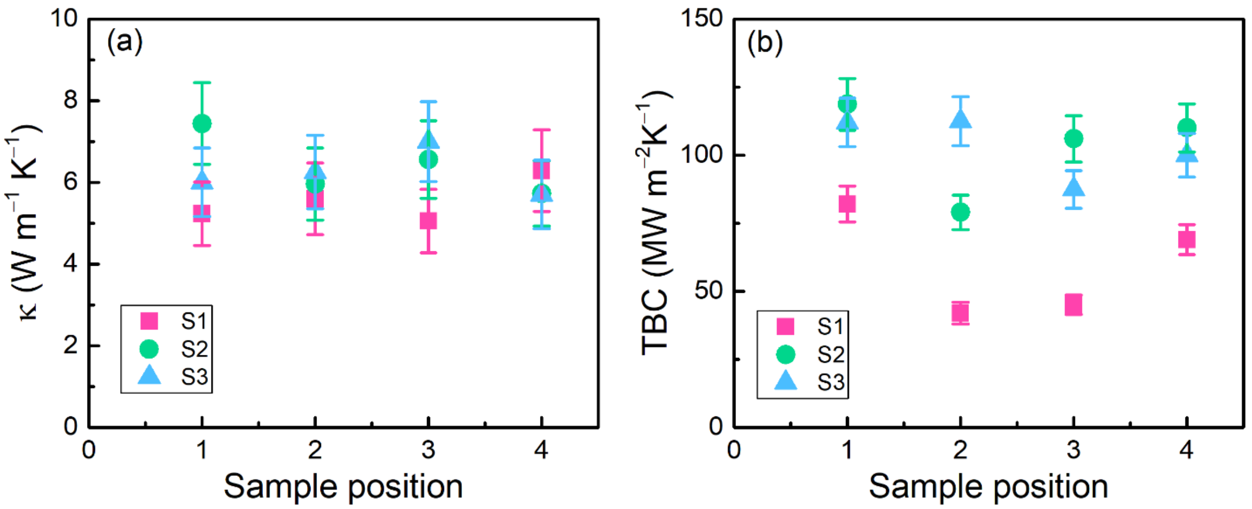

Figure S4. Spatial variation in (a) thermal conductivity (κ) and (b) thermal boundary conductance (TBC) at different measurement locations for untreated (S1) and Ar plasma-treated (S2 and S3) β-Ga_2O_3 samples, measured using TDTR. Error bars represent the fitting uncertainties of individual measurements.

Higher−magnification cross−sectional BF−TEM images of the 1100 and 2100 nm β−Ga_2O_3 films reveal additional microstructural heterogeneity within the thicker layers as shown in Fig. S5. In both samples, bright cavity−like regions are observed predominantly along intergranular boundaries and are attributed to growth-induced voids or intergranular porosity. These features are accompanied by pronounced diffraction contrast associated with the granular/columnar microstructure. The presence of such void−like defects indicates incomplete grain coalescence during film thickening and provides an additional source of structural disorder that may contribute to phonon scattering and the suppression of thermal conductivity relative to bulk β−Ga_2O_3.

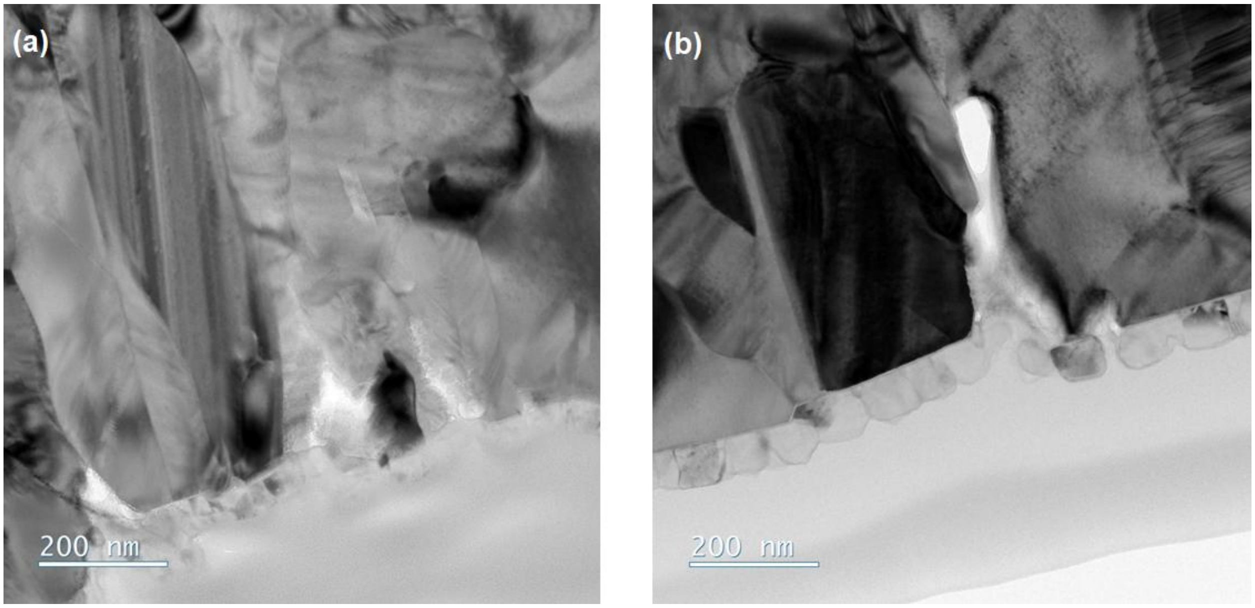

Figure S5. Higher−magnification cross−sectional bright−field transmission electron microscopy (BF−TEM) images

of the (a) 1100 nm and (b) 2100 nm β-Ga_2O_3 films. Bright cavity−like regions located mainly along intergranular boundaries indicate the presence of growth−induced voids or intergranular porosity within the thicker films.

The surface morphology of the β−Ga_2O_3 films was further examined by SEM, as shown in Fig. S6. The 420 nm film exhibits a relatively fine and uniform surface morphology, with characteristic lateral features on the order of 100–200 nm as shown in Fig. S6 (a). With increasing thickness to 1100 nm, pronounced coarsening is observed, accompanied by the formation of larger, strongly faceted and elongated surface features. The 2100 nm film also exhibits a well-developed faceted morphology, although the characteristic lateral feature size is somewhat smaller and the surface appears more densely structured than that of the 1100 nm. Overall, the SEM observations reveal substantial thickness−dependent evolution of the film morphology, with pronounced coarsening from 420 to 1100 nm followed by a more densely faceted surface at 2100 nm. These observations are consistent with the AFM and cross-sectional TEM results in the main text.

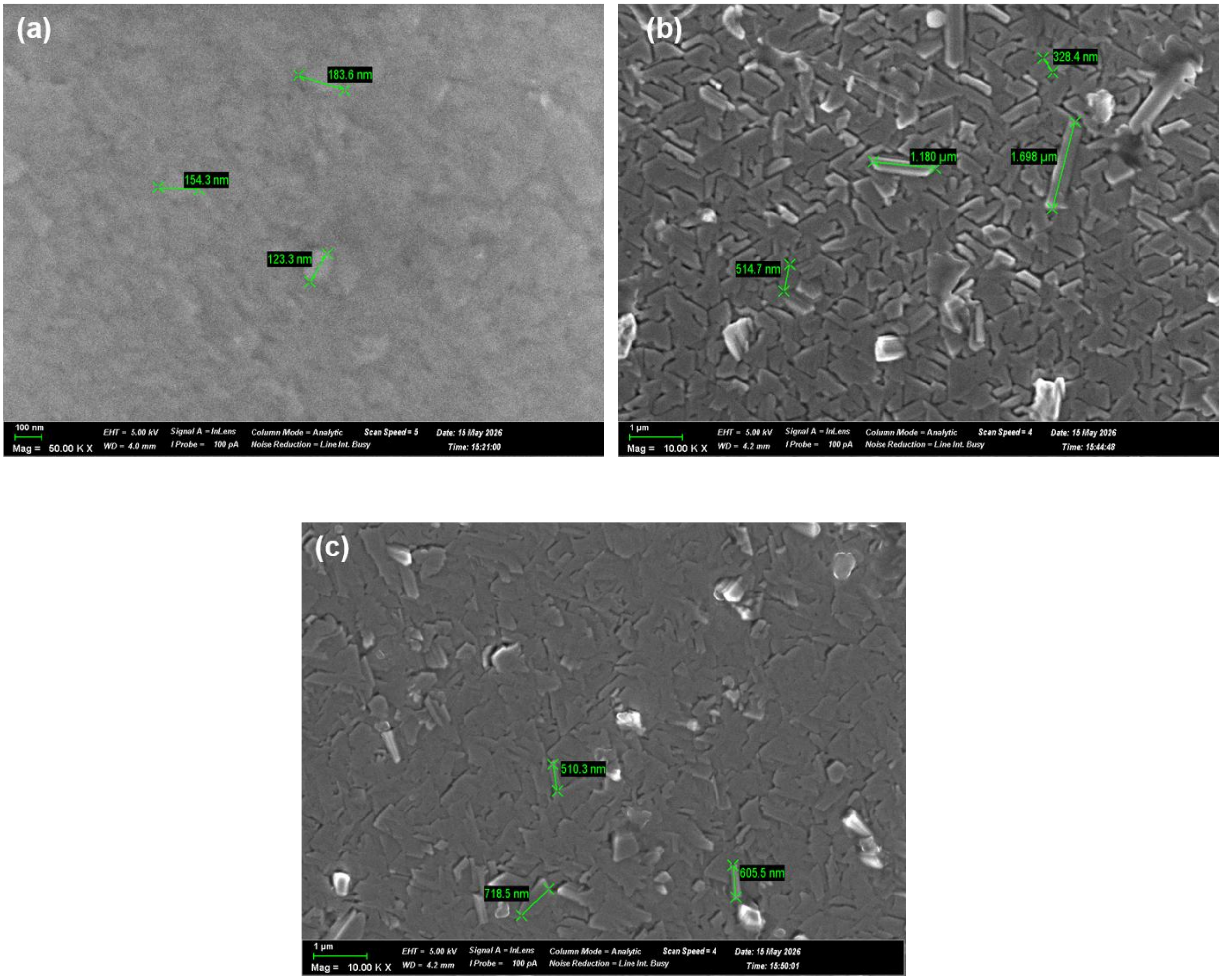

Figure S6. Surface morphology of RF−magnetron−sputtered β−Ga_2O_3 films with thicknesses of (a) 420 nm, (b) 1100 nm, and (c) 2100 nm observed by scanning electron microscopy (SEM).

Supplementary references

12. Zhang, Y.; Su, Q.; Zhu, J.; Koirala, S.; Koester, S.J.; Wang, X. Thickness-dependent thermal conductivity of mechanically exfoliated β-Ga_2O_3 thin films. Appl. Phys. Lett. 2020, 116, 202101.
23. Jiang, P.; Qian, X.; Yang, R. Tutorial: Time-domain thermoreflectance (TDTR) for thermal property characterization of bulk and thin-film materials. J. Appl. Phys. 2018, 124, 161103.
26. Shamatova, A.; Abdullaev, A.; O'Connell, J.; Kušnír, J.; Grabec, T.; Sekerbayev, K.; Skuratov, V.; Utegulov, Z. Anisotropic thermal transport in robust crystalline yttrium aluminium garnet tunable by long oriented amorphous nano-tracks. Int. J. Therm. Sci. 2026, 222, 110583.
27. Cahill, D.G. Analysis of heat flow in layered structures for time-domain thermoreflectance. Rev. Sci. Instrum. 2004, 75, 5119–5122.
28. Schmidt, A.J.; Cheaito, R.; Chiesa, M. A frequency-domain thermoreflectance method for the characterization of thermal properties. Rev. Sci. Instrum. 2009, 80, 094901.
47. Adams, G.B., Jr.; Johnston, H.L. Low-temperature heat capacities of inorganic solids. XI. The heat capacity of β-gallium oxide from 15 to 300 K. J. Am. Chem. Soc. 1952, 74, 4788–4789.
48. Ditmars, D.A.; Ishihara, S.; Chang, S.S.; Bernstein, G.; West, E.D. Enthalpy and heat-capacity standard reference material: Synthetic sapphire (α-Al_2O_3) from 10 to 2250 K. J. Res. Natl. Bur. Stand. 1982, 87, 159–163.
49. Buyco, E.H.; Davis, F.E. Specific heat of aluminum from zero to its melting temperature and beyond. Equation for representation of the specific heat of solids. J. Chem. Eng. Data 1970, 15, 518–523.
